# Are We Using the Right Approach to Change Newborn Care Practices in the Community? Qualitative Evidence From Ethiopia and Northern Nigeria

**DOI:** 10.9745/GHSP-D-19-00410

**Published:** 2020-09-30

**Authors:** Zelee Hill, Pauline Scheelbeek, Yashua Hamza, Yared Amare, Joanna Schellenberg

**Affiliations:** a University College London, London, UK.; b London School of Hygiene & Tropical Medicine, London, United Kingdom.; c ChildCare and Wellness Clinics, Abuja, Nigeria.; d Consultancy for Social Development, Addis Ababa, Ethiopia.

## Abstract

Changing behaviors is usually a core component of the role of community health workers (CHWs), but little is known about the mechanisms through which they change behavior. We collected qualitative data from 8 sites in Ethiopia and northern Nigeria where CHWs were active to understand how they change newborn care behaviors. In each country, we conducted 12 narrative interviews and 12–13 in-depth interviews with recent mothers and 4 focus group discussions each with mothers, fathers, grandmothers, and CHWs. We identified 2 key mechanisms of behavior change. The first was linked to the frequency and consistency of hearing messages that led to a perception that change had occurred in community-wide behaviors, collective beliefs, and social expectations. The second was linked to trust in the CHW, obligation, and hierarchy. We found little evidence that constructs that often inform the design of counseling approaches, such as knowledge of causality and perceived risks and benefits, were mechanisms of change.

## Abstract (precis)

In Ethiopia, high community-level exposure to consistent messages and the perceptions of community health workers and relationships with them drove newborn care behavior change. In Nigeria, exposure to messages was limited, community health workers were less trusted, and behavior change was reported less frequently.

## Teaser

**Key Findings:**

- In Ethiopia, high community-level exposure to consistent messages and the perceptions of community health workers (CHWs) drove behav-ior change.
- In Nigeria, exposure to messages was limited, CHW were less trusted, and behavior change was reported less frequently.

**Key Implications:**

- The role of saturation and trust in behavior change in Ethiopia suggests that CHW counseling interventions should not be assumed to work through didactic provision of messages that focus on the behavior benefits.
- In settings with low coverage of CHW visits, low levels of message reinforcement from other sources, and low trust in CHWs, achieving behavior change may be difficult, even among people who receive visits.

## INTRODUCTION

Community health workers (CHWs) are central to efforts to reduce neonatal mortality in many low-income settings,^1^ and prenatal or postnatal home visits by CHWs are a key component of their role.^2^ A meta-analysis of 8 cluster-randomized studies of home visits by CHWs reported that such visits could reduce neonatal mortality by 11% (risk ratio 0.89; 95% confidence interval [CI]=0.85, 0.94), with the reduction being greater in settings with high mortality and low facility delivery rates.^1^ Although CHWs vary regarding payment and training,^3^ changing caregiver behaviors is usually a core component of their role.

In many cases, CHWs facilitate behavior change through the use of counseling cards that include written instructions for the CHW and pictures to help families understand and remember the benefit of the behavior.^4^ The mother is usually the target of the visits, but including other family members is often encouraged. The counseling approach often focuses on information-centric interpersonal communication, and it is informed by theoretical models developed in high-income settings.^5^^,^^6^ These theories are driven by constructs such as rationality, autonomy, self-interest, and material progression. This approach is exemplified in a recent review of caregiver behavior change in low- and middle-income countries that described the need for caregivers to know about the causal link between the behavior and survival, perceive that a risk exists, experience self-efficacy, have the resources to overcome environmental constraints, and decide to engage in the beneficial practice.^7^ It has been argued that in some low-income settings constructs such as interdependence, relationships, trust, obligation, and collective decision making may be more salient.^5^^,^^8^^,^^9^ There have been calls for more research to understand the mechanisms through which CHW interventions change behavior—the inner workings of the system—so they can be better designed and targeted.^5^^,^^10^^–^^12^

Current evidence suggests that CHWs are much more than conduits of information. A common theme among studies exploring how CHWs effect change is that they can serve as brokers and connectors.^11^^,^^13^^–^^16^ They achieve this through connecting people with services, using local networks,^13^ engaging with influencers,^12^^,^^17^ and advocating for external resources or appealing to authorities.^17^^–^^19^ Having an empathetic, caring, and trusting relationship with the community is considered fundamental to the workers’ success.^11^^,^^14^^,^^15^^,^^20^^–^^23^ Trust comes from CHWs being embedded in the community—that is, an “insider”—whom community members identify with and have confidence in.^13^^,^^14^ Being an insider also means that CHWs understand and are direct witnesses to communities’ needs and challenges and are well placed to know how to address them.^12^^,^^13^^,^^24^ CHWs can engage with community members and build rapport with them; use appropriate terms to talk about health (e.g., through local idioms or songs); use their own personal testimonies of behavior change or act as role models; package benefits to be appealing and salient to community members; and identify relevant strategies for behavior change such as persistence and repetition.^11^^–^^14^^,^^17^^,^^19^^–^^22^^,^^24^^,^^25^ CHWs have also been seen to affect change by taking on out-of-scope tasks or using personal resources to facilitate their role.^11^^–^^13^^,^^17^^,^^21^^,^^22^^,^^25^

Current evidence suggests that CHWs are much more than conduits of information.

CHWs also differ from other community members due to their training, skills, and association with the health system, which places them as “outsiders.” As such, they can have increased acceptance, respect, and status.^13^^,^^14^^,^^21^^,^^23^^,^^24^ Trust in CHWs as outsiders can be part of a broader system of hierarchical trust in authorities,^21^ which may result in community members feeling obliged to follow advice from CHWs^26^ and may reduce a community’s ability to make informed decisions.^21^

Trust and credibility can be lost if CHWs do not meet community expectations, for example, if they lack supplies or demonstrate poor capabilities or when communities doubt their motives.^11^^,^^13^^,^^16^^,^^23^ Motives can be questioned when CHWs are perceived as not working for the interest of the community but as having alliances elsewhere,^16^ either because of the selection process^22^ or because there is a widescale distrust of authorities.^22^

In this article, we report data from both CHWs and the community, exploring how CHWs change newborn care behaviors in Ethiopia and northeast Nigeria. We use an approach informed by realist evaluation to identify the mechanisms and the contextual factors that influence change.^27^ In Ethiopia, we focus on health extension workers (HEW) and the Health Development Army (HDA) leaders who were supported by the Last Ten Kilometers (L10K) program, and we compare our findings with those from northeast Nigeria around the role of community volunteers supported by the Society for Family Health (SFH).

## METHODS

### Study Setting Selection and Characteristics

Data were collected between March and June 2015 in the Southern Nations, Nationalities, and Peoples Region and the Amhara region in Ethiopia and in Gombe state in Nigeria. Table 1 shows the key characteristics of the CHWs in both sites. In Ethiopia, HEWs underwent 1 year of training, were paid about $100 a month, and served around 5,000 people. They provided health promotion and disease prevention and treatment, and they worked both at their health post and in the community.^28^^,^^29^ In Gombe, Nigeria, the CHWs were women from the Federation of Muslim Women’s Association in urban areas and traditional birth attendants in rural areas. They underwent 5–6 days of training and were volunteers, but they received incentives for accompanying women to facilities for delivery and for referring women with danger signs.^30^

**TABLE 1.** Characteristics of Community Health Workers in Nigeria and Ethiopia

	**Nigerian CHWs**	**Ethiopian HEWs**	**Ethiopian HDA leaders**
Training	5–6 days	1 year	15 days
Payment	Incentives for taking women to facilities and for referrals	Approximately $100/month	None
Selection criteria	Existing TBAs or FOMWAMs, no literacy requirement	Resident in the community, speaks local language, educated to 10th grade or above, willing to remain in the village and serve the community	Model family, trusted, able to mobilize communities; no literacy requirement
Scope of work	Specific to maternal and newborn health including delivering key behavior change messages, detection of and referral for maternal and newborn danger signs	Broad, including disease prevention and treatment	Broad, including assisting families in adopting behaviors, engaging in community mobilization, and leading participatory action cycles

Abbreviations: CHW, community health worker; HDA, Health Development Army; HEW, health extension worker; FOMWAM, Federation of Muslim Women’s Associations in Nigeria; TBA, traditional birth attendant.

In both the Ethiopian and Nigerian sites, CHWs made prenatal and postnatal home visits, which included promoting neonatal care practices and service utilization. In Ethiopia, the HEWs also provided counseling during antenatal care at health posts, and all women were part of the HDA. The HDA members were meant to work in small groups with a leader whose role included helping families adopt the HEW messages by identifying pregnant women and linking them to services, holding monthly meetings, and running participatory learning and action cycles. The HDA leaders were selected by their group, were volunteers, and received an average of 15 days of training facilitated by the HEWs.^31^^–^^34^

With the assistance of L10K and SFH program staff, we identified 4 study sites in each country that had CHWs in place who were considered to be active. In Ethiopia, we selected 4 *kebeles* (districts) that had no unusual characteristics such as being near an industrial center or a tertiary hospital. In Nigeria, we selected 4 local government areas (LGAs) (districts)—2 urban and 2 rural. Insurgents were active at the time of data collection, which meant that the study team needed to return to the state capital each day, and LGA selection was limited to sites that were within a few hours drive of the state capital. In Ethiopia, data collectors were able to access study sites that were more remote. Table 2 shows the characteristics of the selected study sites. The access to health care described in the Table was determined by the fieldworkers based on distance to the nearest health centers, availability of transport, and the general road conditions.

**TABLE 2.** Characteristics of Study Sites in Ethiopia and Nigeria

**Region**	**Study area**	**Ethnicity**	**Main Religions**	**Access to Health Care**	**Terrain**	**Main Economic Activities**
Amhara, Ethiopia	Kebele A	Amhara	Orthodox Christian	Moderate	Hilly	Subsistence farming
	Kebele B	Amhara	Orthodox Christian	Good	Hilly	Subsistence farming
SNNPR, Ethiopia	Kebele C	Gamo/Wolaita	Protestant/Orthodox Christian	Good	Flat with some hills	Subsistence farming
	Kebele D	Silte	Muslim	Moderate	Flat	Subsistence farming
Gombe, Nigeria	LGA A: urban	Tangle	Christian	Excellent	Flat	Traders, farmers
	LGA B: rural	Tangle	Christian	Good	Flat	Traders, farmers
	LGA C: urban and close to capital	Tera, Bolewa, Fulani	Muslim	Excellent	Flat	Traders, civil servants, farmers
	LGA D: rural and close to capital	Fulani, Kanuri	Muslim	Excellent	Flat	Traders, civil servants, farmers

Abbreviations: LGA, local government area; SNNPR, Southern Nations, Nationalities and Peoples Region.

### Behaviors

Interventions that have the greatest impact on neonatal mortality are those that improve care in labor, during birth, in the first week of life, and for small and sick babies.^35^ Interventions to improve immediate newborn care such as thermal care (drying and wrapping, skin-to-skin care, delayed bathing), hygienic cord care, and early initiation of breastfeeding are central to these interventions,^36^ and CHWs often promote these behaviors. To explore behaviors in sufficient depth, we focused on thermal care and breastfeeding behaviors. Specifically, we focused on the following behaviors: drying the newborn and either placing it in skin-to-skin contact with the mother or wrapping the newborn after delivery if skin-to-skin contact was not possible; delayed bathing; and immediate and exclusive breastfeeding.^37^

Interventions having the greatest impact on neonatal mortality are those that improve care in labor, during birth, in the first week of life, and for small and sick babies.

### Interview and Focus Group Discussion Guide Development

The interview guides included free-flowing questions, such as a description of labor, delivery, and newborn care, to allow unanticipated themes to emerge (Table 3). These questions were followed by theory-driven questions identified from the study theoretical framework (Figure), which hypothesizes a set of mechanisms through which CHWs could change neonatal breastfeeding and thermal care behaviors, and the contexts that may enable or constrain these mechanisms. The framework was developed using a realist evaluation approach,^27^ the COM-B (capability, opportunity, motivation, and behavior) model,^38^ a review of behavior change theories from a range of disciplines,^39^^–^^42^ a systematic review of neonatal care practices in sub-Saharan Africa,^43^ and discussions with implementers in the 2 countries. All guides were pretested and amended as needed.

**TABLE 3.** Data Collection Method, Sample Size per Country, and Content in Ethiopia and Nigeria

**Method**	**Sample**	**Content**
Narrative interviews with recent mothers	12	Labor and delivery story How was the newborn cared for, by whom, what influenced care, who made decisions Perceived newborn care knowledge and skills compared with others Contacts with, and advice from, health workers and CHWs, was advice new, did they agree with it, did it influence their behavior Other advice received, agreement with the advice Importance of what family and friends think of the care they give
In-depth interviews with recent mothers	12–13	Newborn care practices in the community and what influences these Influence and importance of family and friends on care CHW roles and their suitability for the role Most significant newborn health changes in last 2 years
FGD with recent mothers	4	Pile sort of feeding and thermal care cards into practiced/not practiced and promoted/not promoted by CHW Completion of a story of conflicting advice about delayed bathing Most significant newborn health changes in last 2 years Reaction to statements that CHWs work does not bring change, and that grandmothers are responsible for newborn care
FGD with grandmothers	4	Reaction to pictures of feeding and bathing practices Role of grandmothers in newborn care and in decision making Ranking of people who influence newborn care Completion of a story of conflicting advice about delayed bathing Most significant newborn health changes in last 2 years Reaction to statements that grandmothers’ role is to support traditional practices, that CHWs know everything about newborn care and that mothers do not listen to grandmothers
FGDs with fathers	4	Reaction to pictures of feeding and bathing practices Role of fathers in newborn care and in decision making Completion of a story of conflicting advice about delayed bathing CHW roles and their suitability for the role Fathers’ knowledge of CHWs’ advice, and the advice they trust most Most significant newborn health changes in last 2 years Reaction to statements that mothers and fathers should decide on newborn care, that CHW visits involve fathers, that CHWs do not bring change, and that grandmothers’ role is to support traditional practices
FGD with CHWs	4	Pile sort of feeding and thermal care behaviors practiced/not practiced and important/not important by CHW Most significant newborn health and work changes in the last 2 years Successes and challenges in their work Community reaction to them and their work Reaction to statements that families are always happy to see the CHW, that CHWs do not bring change, and that families agree with delayed bathing advice

Abbreviations: CHW, community health worker; FGD, focus group discussion.

**FIGURE.**
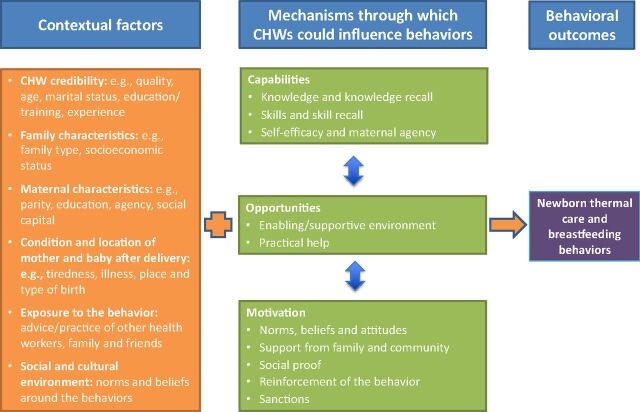
Hypothesized Mechanisms Through Which Community Health Workers Could Affect Neonatal Care Practices and the Contexts that May Influence the Mechanisms in Ethiopia and Nigeria Abbreviation: CHW, community health worker.

### Data Collection

Narrative and in-depth interviews (IDIs) were conducted with mothers at their houses. Focus group discussions (FGDs) were conducted with mothers, grandmothers, fathers, and CHWs in a neutral location, such as a school for community members and the health centers for CHWs. Having multiple respondent groups allowed us to capture different perspectives and to triangulate findings. We conducted narratives with mothers to understand their experiences and to see how events influenced each other and IDIs to capture perceptions. FGDs were conducted with 3–7 participants each to explore issues that would benefit from being discussed. We enhanced FGD interaction and reduced social desirability bias through activity-oriented exercises.^44^ Interviews and FGDs lasted between 45 minutes and 2.5 hours.

Community respondents were identified through community informants, such as women’s organization leaders, by the CHWs, at places of worship, and through snowball sampling. As we were interested in the mechanisms through which CHWs change behavior, we excluded respondents who had no contact with these cadres. In Ethiopia, due to the small numbers of HEWs in a *kebele*, all HEWs in the study *kebele* and those from neighboring *kebeles* were invited to participate in the FGDs. The SFH CHWs in Nigeria were identified with assistance from SFH and through snowball sampling. For mother interviews, we aimed to get a range of participants regarding age, educational level, parity, sex of newborn, and place of delivery.

Data were collected in local languages by 4–6 interviewers in each country. In Ethiopia, interpreters were occasionally needed to translate from Amharic into Silte. Interviewers were social science graduates with 2–17 years of qualitative research experience. They received 4 days of classroom training, which included a detailed review and discussion of the content of the guides, probing strategies, transcription, and ethics. This training was followed by a 2-day field pilot.

Interviewers approached potential community respondents in their homes and CHWs at their homes or health posts, explained the study, obtained written informed consent, and arranged a convenient time for the interview. Three respondents declined to be interviewed, stating that they were too busy. Interviews and FGDs were audio recorded, transcribed, and translated into English by the interviewers during data collection with conceptual and, where possible, semantic equivalence.^45^

Data collection stopped when additional interviews and FGDs did not provide new information. This point was determined by frequent transcript reviews and through discussions with interviewers. The sample size, respondent groups, and the interview content are shown in Table 2.

Ethical approval was granted by the Ministry of Science and Technology in Ethiopia, the National Research Ethics Committee and the Gombe State Government in Nigeria, and the London School of Hygiene & Tropical Medicine in the United Kingdom.

### Data Analysis

Analysis began during data collection through regular team meetings and 2 data analysis workshops to discuss emerging themes, receive feedback on transcripts, remove or add questions, and increase reflexivity. These initial themes and the theoretical framework were used to develop a deductive coding template in Nvivo. Interviews and FGDs were then coded inductively within these broad themes, with new themes added as they emerged. Coding and interpretation were done separately for each country by 4 of the senior researchers (ZH, YH, YA, and PS), who initially read through the transcripts to identify a first set of inductive themes and codes and to get an impression of the data as a whole. To enhance coding rigor and conceptual thinking, initial coding was done through consensus coding of the same transcripts, a code book was developed, and the team regularly met to discuss coding. Each analyst then took a set of transcripts to code. The study lead author (ZH) checked all coding using the coding stripe function of Nvivo. Coding discussions focused around merging conceptually similar codes and examining patterns, links, and contradictions in the data. We triangulated findings between respondent groups and data collection methods to check credibility. Due to logistical constraints and literacy issues, we were unable to return transcripts to participants for comment.

## RESULTS

Table 4 shows the characteristics of the narrative and IDI respondents. Respondents had a range of ages, education levels, parities, and religion. We did not achieve the planned diversity in place of delivery possibly because we were working in more accessible areas (close to the state capital in Gombe state and within walking distance of a motorable road in Ethiopia), in areas where CHWs were active, and with women who had received at least 1 visit by a CHW. In addition, CHWs assisted in identifying some respondents and may have favored those who delivered in a facility.

**TABLE 4.** Sample Characteristics of Mothers in Narrative and In-Depth Interviews in Ethiopia and Nigeria

**Characteristic**	**Ethiopia (N=25)**	**Nigeria (N=24)**
Age, years		
≤24	10	8
25–34	10	12
≥35	5	4
Education		
None	10	12
Primary	12	4
Secondary and above	3	8
Religion		
Muslim	8	15
Christian	17	9
Parity		
1	7	3
>1	18	21
Place of last delivery		
Home	6	4
Facility	19	20
Residence		
Urban	0	12
Rural	25	12

The FGD participant mothers varied in age (range 20–40 years), parity (range 1–8 children), education (none to secondary level), and ethnicity. The FGD participant fathers were older (range 28–61 years of age), and the FGD participant grandmothers were almost all uneducated.

Most families interviewed in Ethiopia reported that they adopted the practices that the HEWs and HDA leaders promoted, and they highlighted that it was a change from their past behaviors:

*In previous time, the baby was bathed immediately after birth, but now, based on the teaching of health post and health center, we wrap the baby immediately after birth.* — Father, FGD Kebele C, Ethiopia

*The previous and the current situations … . are like the earth and the sky.* —Father, FGD Kebele C, Ethiopia

Most families interviewed in Ethiopia reported adopting practices promoted by the HEWs and HDA leaders, which differed from past behaviors.

Skin-to-skin care was the only thermal care practice that was reported as not being promoted, and this practice was new to most of the community respondents.

We identified 2 interlinked mid-range theories relating to how HEW interactions with families influenced the promoted practices in Ethiopia—*saturation* and *trust* theories. A third theory, *facility behavior*, was identified but applied only in the context of women who delivered in a facility.

The saturation theory describes a set of mechanisms that relate to families receiving consistent messages, reinforced by multiple sources (HEW, HDA leaders, and health workers) at multiple time points (before pregnancy, pregnancy, and at delivery) and through multiple channels (community meetings, home visits, antenatal care, and at delivery):

*We are told not to give anything until 6 months . … [By] [HDA leader] [HEW], and we are told in the health center or when we go to the health post for vaccination [antenatal care].* —Mother, IDI, Kebele D, Ethiopia

*I heard this advice before, but when they give me additional advice it strengthen the idea…the HEWs advised me that the baby should not be bathed immediately after birth, and it was the same thing that the health professionals advised me.* —Mother, IDI, Kebele A, Ethiopia

This saturation of messages led to high levels of community-wide familiarity with the promoted behaviors, with social diffusion further reinforcing the messages:

*I didn’t hear that with my ears but I heard a rumor that if you delivered at health facility you have to bathe [the baby] after 3 days.* —Mother, Narrative 1, Kebele D, Ethiopia

Saturation of messages led to high levels of community-wide familiarity with promoted behaviors, with social diffusion further reinforcing the messages.

This familiarity led to a perception that there had been a change in collective beliefs and practices and a social expectation that the new behaviors should be practiced:

*No one [in the family or community] suggested bathing the baby immediately … They all know that the baby should be bathed after spending a day.* —Mother, Narrative, Kebele C, Ethiopia

*It is the same [people’s behavior], it has no difference. They [others] have received the lesson . … They have received the same lesson as me.* —Mother, Narrative, Kebele C, Ethiopia

The HEWs attributed message penetration to the formation of the HDA, whose members were able to have more frequent contacts with households. This attribution was confirmed by some community respondents.

Having messages provided from multiple sources meant that even if a worker was less active, messages were still heard. For example, in 1 study site, HEWs were reported as being relatively inactive in making home visits, but most women still received messages from other sources.

Grandmothers were also familiar with the promoted behaviors and, with the exception of delayed bathing, were generally supportive of them, recognizing that this was a new time:

*It is our children who advise us about what they know … Today’s mothers are wise. In previous time, there was no education.* —Grandmother, FGD, Kebele D, Ethiopia

Grandmothers were also familiar with the promoted behaviors and were generally supportive of them.

As a result of being “educated” about the practices, mothers had increased maternal agency and felt more knowledgeable than their “outdated” elders and more able to reject their advice:

*These days, they [HEW] give us good education on how to take care of babies … my families are getting older … So even though they give us advice, we would not follow that … I follow my own way.* —Mother, Narrative, Kebele B, Ethiopia

Overall, fathers were less familiar with the details of the behaviors being promoted, but they were supportive of the CHWs and health worker advice, as illustrated in this conversation in one FGD:

*Previously … . our mothers decided.* —Father A, FGD, Kebela A, Ethiopia

*Now I can decide that it [food] should not be given before 6 months.* —Father B, FGD, Kebele A, Ethiopia

*Because health professionals teach this.* —Father A, FGD, Kebela A, Ethiopia

Familiarity with the messages was important, but knowledge of the benefit of the behavior was not a prerequisite for adoption. For example, although many respondents could report that delayed bathing helped maintain warmth, several did not know the reason for the practice but still reported that they carried it out. In these cases, the trust theory was the driver of change:

*I do what they told me to do; I waited the time and bathed the baby … I was thinking it could not be harmful; they said this because it is useful. —*Mother, IDI, Kebele D, Ethiopia

Familiarity with the messages was important, but knowledge of the benefit of the behavior was not a prerequisite for adoption.

The trust theory relates to a trust in the CHWs and health workers as a collective to act in the community’s interest, leading families to follow the advice given:

*They [mothers] listen to the HEW, even if they tell you that you have to walk naked.* —Grandmother, FGD, Kebele C, Ethiopia

Trust was related to CHWs’ high status in that they were “better” and “more educated” than community members:

*I was advised about it before delivery. I accept what they told me because they know better. —*Mother, Narrative, Kebele C, Ethiopia

*We just respect educated people. They are educated, and we are just farmers.* —Mother, IDI, Kebele A, Ethiopia

Trust was also related to CHWs being seen as connected to the government, as well as to their visibility, connectedness, and understanding of the community.

Although the HEWs as a collective were trusted as acting in the community’s interest, this trust could be lost for individual HEWs, for example, if they provided inequitable services, had a poor work ethic, took bribes, or were unkind:

*They [HEWs] are not responsible enough to carry out their tasks … they want you to give them something as a bribe … . if she doesn’t give something as a bribe, the woman won’t get any service … they did not have good manner.* —Mother, IDI 5, Kebele B, Ethiopia

Trust could be lost for individual HEWs if they provided inequitable services, had a poor work ethic, took bribes, or were unkind.

Trust in the HEWs and their status were combined with a hierarchical relationship that resulted in families feeling obligated to follow the advice of HEWs. This circumstance is illustrated by community respondents describing behaviors as “forbidden” or “not allowed” and reporting that the HEWs “commanded” them. This hierarchical relationship was compounded by a belief that the HEWs had the power to make life difficult if advice was not followed, either by being angry with families or denying them services.

HDA leaders did not have the same status as HEW/health workers and were described as “villagers just like us.” They were viewed as conduits of information and were important drivers of the saturation mechanisms but were not drivers of the trust mechanisms:

*They give advice as local people, not different from advising as a neighbor.* —Father, FGD, Kebele B, Ethiopia

The third theory, centered on facility behavior, relates to the recent increase in facility deliveries, which meant that some family practices had become health worker practices:

*When they deliver at the health center, they practice this [immediate breastfeeding] … they practice it mostly because they deliver at the health facility.* —CHW, FGD, Kebele A Ethiopia

At a facility delivery, immediate drying and wrapping were done by the health workers, who also could insist on early breastfeeding and no bathing during the stay in the facility.

The 3 theories described above were not triggered equally for all behaviors, with delayed bathing being the most difficult behavior to change. This was related to some families, and in particular grandmothers, having strong culturally entrenched beliefs against leaving the baby “dirty” after birth. This was especially the case if the baby was bloody or had an obvious vernix:

*We will bathe the baby anyway, how can we sit idle without bathing the baby.* —Mother, IDI, Kebele D, Ethiopia

Another contextual factor that enhanced the uptake of behaviors was the value given to education and modernity:

*Do you think I want to do like in the past? I want it to be just like the modern time … Today’s mothers are young and modern. They easily accept new ideas.* —Mother, Narrative Kebele C, Ethiopia

In the Nigerian sites, there were fewer reports of behaviors being changed, and the impact of saturation was less striking than in Ethiopia, as messages were received less often and from fewer sources. There was less evidence of a change in collective beliefs as well as more cultural barriers to behavior change, especially when mothers received conflicting advice:

*… I gave water on the third day … I am confused with the advice I am getting, some say give, some say do not give.* —Mother, Narrative, LGA C, Nigeria

In the Nigerian sites, there were fewer reports of behaviors being changed, and the impact of saturation was less striking than in Ethiopia.

As in Ethiopia, CHWs were generally trusted in the Nigeria sites, but fewer respondents reported that they carried out behaviors without knowing their benefits:

*I’ll follow the ways that I already know, I’ll follow the old ways until I hear more explanation on it.* —Mother, Narrative, LGA C, Nigeria

As in Ethiopia being “educated” by a CHW allowed some mothers to challenge the advice of elders, and a theme in the grandmother FGDs emerged around times changing and their advice no longer being relevant:

*We the old grandmothers are in trouble. We have been swept into the dustbin.* —Grandmother, FGD, LGA A, Nigeria

But in many households, elders remained key influencers of newborn care with a strong family hierarchy. For example, several mothers reported they followed or pretended to follow the advice of the elders to ensure harmonious relationships.

In many households, elders remained key influencers of newborn care, with a strong family hierarchy.

Unlike in Ethiopia, the facility behavior theory was not a key mechanism of change in Nigeria, as health staff did not practice the behaviors that CHWs were promoting as frequently as in Ethiopia:

*At hospital when you deliver, you will be allowed to rest for 1 hour … which in most cases, you will be sleeping. So, you see you don’t even have the time to breastfeed your baby*. —Mother, IDI, LGA D, Nigeria

However, a theme emerged around a facility delivery giving women more autonomy in decision making because they were viewed as having adopted and being exposed to modern practice:

*She [grandmother] used to say I should breastfeed them, and about water … I should give them … I tell her that in the hospital they told us not to give and she will not force me, she will respect what the hospital says.* —Mother, Narrative, LGA A, Nigeria

As in Ethiopia, the mechanisms were not triggered for all behaviors in Nigeria, but in contrast to Ethiopia, delayed bathing was more amenable to behavior change than breastfeeding practices. This was because families had been convinced that the baby could be cleaned adequately by rubbing them with oil rather than bathing them, making delayed bathing culturally acceptable:

*They [health worker] clean the baby very well, so baby will not smell, if they leave the baby dirty … then, of course the baby will smell, my baby was very clean so, no problem.* —Mother, Narrative, LGA B, Nigeria

## DISCUSSION

The saturation and trust theories that we identified in our study sites are similar to findings from previous studies on how CHWs affect change,^11^^,^^14^^,^^15^^,^^17^^,^^20^^–^^23^ but are in contrast to the idea that knowledge of a causal link and perception of a risk are needed for behavior change.^7^ Many behavior change theories include constructs related to psychosocial, environmental, and social network determinants of behaviors,^46^ yet there can be an overreliance on didactic communication in health promotion.^47^

The saturation mechanism was linked to frequent and consistent information, which was provided more often in Ethiopia than in Nigeria. Where it occurred, saturation led to a perception that there had been a change in community-wide behaviors, collective beliefs, and social expectations of behaviors. CHW counseling interventions usually focus on changing the behavior of one individual at a time, with less emphasis given to message saturation or exposure.^48^^,^^49^ CHWs themselves have identified the need for persistence and repetition,^11^^,^^17^ and when sufficient in number, positive testimonies from community members about CHWs’ work have been found to create a snowball effect and enhance community-wide trust.^21^

In the Ethiopian sites, high exposure to consistent information was achieved, but obtaining the same outcome may be difficult in many settings. Few countries have reached more than 10% coverage for postnatal home visits,^50^ and some evidence suggests that in some settings, CHWs lacked the time, motivation, and support to engage with high-quality behavior change.^19^ Behavior change may be difficult to achieve in settings with less functional CHW programs, which may in some part explain the difference in the findings between Ethiopia and Nigeria. This difference highlights the importance of context in the design of counseling interventions within CHW programs.

The trust mechanism was related to trust in CHWs, status, obligation, and hierarchy but also to CHWs’ closeness to and understanding of the communities they serve. In Ethiopia, this mechanism was seemingly more important than individuals being convinced of the specific benefit of a behavior. Previous studies have highlighted the importance of relationships, trust, and power between CHWs and the community, and the impact that these factors can have on acceptability, motivation, and performance.^11^^,^^13^–16^,^^20^^–^^24^^,^^26^^,^^51^^–^^53^ We demonstrate the centrality of this relationship to behavior change, and a greater emphasis on it may be warranted in programs. The trust mechanism was likely also influenced by the unique Ethiopian social and political context at the time of the study, which was characterized by a stratified and hierarchical society with strong political control and will^54^ and high levels of social cohesion.^55^ The role of hierarchical trust may be particularly strong in Ethiopia compared with Nigeria and other settings, which may have resulted in caregivers not making informed decisions but feeling obligated to comply with the CHWs advice.^21^

In the Nigerian sites, respondents reported that messages were received less often and from fewer sources. In addition, the advice of CHWs was questioned more, and elders remained as key influencers. These findings could be attributed to a poorer functioning CHW system in Gombe, with lower coverage than in Ethiopia,^56^^,^^57^ and the lower training and the volunteer nature of the CHWs, as well as the broader cultural, political, and health systems contexts. For example, in Ethiopia, the HEWs provided services both in the community and at the health post, which may have enhanced their status and their ability to influence the community compared with the CHWs in Nigeria who only had a community role. In addition, consistent messaging may have been harder to achieve in Nigeria, which was characterized by interventions with disjointed designs, a proliferation of strategies, and multiple health systems, which created unique challenges.^58^^–^^62^ Further, there may have also been less hierarchical trust in authorities.

### Limitations

We used multiple study sites, purposive sampling to saturation, reflexivity, triangulation of methods and respondent groups, consensus coding, and within and across case analysis to increase data quality and the transferability of findings.^63^ However, the findings may not be transferrable to settings with very different contexts, especially those that are more remote and inaccessible. This limitation is particularly the case in Nigeria, where our site selection was restricted by insurgent activities. The inclusion of more women who delivered at home would have allowed us to examine differences in mechanisms by place of delivery. The potential exists that reporting was influenced by social desirability bias, a particular problem in Ethiopia.^54^ In addition, the respondents identified by CHWs may have had different attitudes and experiences compared with families that were perhaps less favored by the CHWs.

## CONCLUSION

Our study did not aim to generalize about the effectiveness of the CHW programs but to uncover the mechanisms through which they may work when they are functioning. CHW programs face issues with inadequate training and support, high workloads, and poor motivation and performance,^3^ and these issues have been found to influence CHWs’ ability to deliver behavior change interventions.^19^ The mechanisms we uncovered are unlikely to be triggered unless health systems function adequately and coverage and quality are improved.

Inadequate training and support, high workloads, and poor motivation and performance can influence CHWs’ ability to deliver behavior change interventions.

## Notes

**Peer Reviewed**

**First published online:** July 24, 2020
